# Does level of training influence the ability to detect hepatosplenomegaly in children with leukemia?

**Published:** 2012-09-30

**Authors:** Donna L. Johnston, Janelle Cyr

**Affiliations:** Children’s Hospital of Eastern Ontario, Ottawa, Ontario, Canada

## Abstract

**Background:**

Children with leukemia often have hepatosplenomegaly present. This can be diagnosed with physical examination and confirmed with ultrasound. We sought to determine if level of training influenced the ability to detect hepatosplenomegaly.

**Methods:**

All children diagnosed with leukemia during the past 5 years were reviewed. The training level of the examiner, the documentation of hepatosplenomegaly, and the ultrasound findings were collected and analyzed.

**Results:**

There were 245 examinations of the spleen and 254 of the liver. Splenomegaly was correctly diagnosed by medical students 54% of the time, by residents 81%, and by staff 79% of the time. First year residents diagnosed it correctly 68% of the time, R2s 64%, R3s 76% and R4s 86% of the time. Hepatomegaly was correctly diagnosed by medical students 44% of the time, by residents 73% and by staff 68% of the time. First year residents diagnosed it correctly 77% of the time, R2s 54%, R3s 81% and R4s 75% of the time.

**Conclusions:**

Pediatric residents had the best ability to detect hepatosplenomegaly, and were better than staff and medical students, although this was not statistically significant.

## Introduction

Children with leukemia often have hepatomegaly and splenomegaly present at the time of diagnosis. Hepatomegaly and splenomegaly can be diagnosed with physical examination and this can be confirmed with ultrasound imaging, which is regarded as the best method to document the size of these organs.^1^–^4^ The detection of hepatomegaly and splenomegaly by physical examination is a skill that is taught in medical school by experienced clinicians. The presence of hepatomegaly and splenomegaly is not a common physical finding in clinical practice, so that often the detection of these abnormalities by trainees may not be accurate due to its rarity. Previous studies have shown that level of training can influence clinical skills, for the most part in a positive way such that skills improve with increasing level of training,^5^–^12^ but there are some studies that show that certain skills decline with increasing level of training,^13^ or that there is no change during training or in practice.^14^–^20^ No studies have previously examined the influence of the level of training on the ability to detect hepatomegaly or splenomegaly.

Hepatomegaly and splenomegaly are rare findings, and we hypothesized that the detection of these abnormalities would be higher in medical students than in residents, because of their proximity to learning this clinical skill. We sought to determine if the level of training influenced the ability to detect hepatosplenomegaly.

## Methods

The charts of all children diagnosed with leukemia at the Children’s Hospital of Eastern Ontario (CHEO) during the past 5 years (2006–2011 inclusive) were reviewed. CHEO is a tertiary academic referral center associated with the University of Ottawa. Children were identified from the Pediatric Oncology Group of Ontario (POGO) database for CHEO, which has detailed information on all children diagnosed with cancer in Ontario. Data collected from these charts included the level of training of the person examining the patient, the documentation of hepatosplenomegaly, the location in the hospital where the examination occurred, and the ultrasound findings. The levels of training were defined as follows: medical student, first year pediatric resident (R1), second year pediatric resident (R2), third year pediatric resident (R3), fourth year pediatric resident (R4) and staff physician. The method of hepatosplenomegaly examination, palpation versus percussion, was not collected so the technique of detection was not assessed. Only children who had an ultrasound examination performed were included in this study, as this was the criterion used to confirm the presence of hepatosplenomegaly found on physical examination. In all cases, the assessments collected were done prior to the child having an ultrasound examination. If the ultrasound findings stated that the spleen or liver was enlarged, then this was taken as proof of splenomegaly or hepatomegaly respectively. Documentation of enlargement was considered positive if the chart stated “enlarged:, “palpable spleen”, “liver below costal margin”, or any other statement indicating enlargement. Data were collected and a descriptive analysis performed.

Statistical analysis was done using SPSS version 19. A *p*-value less than 0.05 was considered statistically significant. The study was approved by the Research Ethics Board at CHEO.

## Results

There were 105 children diagnosed with leukemia during the study time period. Of these, 94 had ultrasound examinations of the liver and spleen performed, and were thus included in this study. The characteristics of these patients are shown in Table 1. Among these 94 children, there were 245 examinations of the spleen and 254 of the liver during the study period. In terms of splenomegaly, there were 36 examinations by medical students, 139 by residents, and 70 by staff. The examinations occurred in the emergency room 85 times, and on the inpatient unit 163 times. Splenomegaly was correctly diagnosed by medical students 54% of the time, by residents 81% of the time, and by staff 79% of the time. This difference in detection based on the clinician’s level of training was not statistically significant (*p* = 0.39). For the residents, first year residents (R1s) diagnosed it correctly 68% of the time, second year residents (R2s) 64%, third year residents (R3s) 76% and fourth year residents (R4s) 86% of the time. This difference also was not statistically significant (*p* = 0.34). In terms of location of diagnosis, splenomegaly was correctly diagnosed 76% of the time in the emergency department and 69% of the time on the inpatient unit (*p* = 0.60). Splenomegaly was under-diagnosed (physical exam noted as normal and ultrasound documented enlarged) in 61 of 245 examinations or 25% of the time. It was over-diagnosed (physical exam noted as enlarged and ultrasound examination normal) in 10 of 245 examinations or 4% of the time. For both splenomegaly and hepatomegaly examinations by staff, in the emergency department these were performed by the emergency room staff, and on the inpatient units by a combination of general pediatricians and pediatric hematologist/oncologists.

Hepatomegaly examinations were performed by 35 medical students, by 147 residents, and by 72 staff. The examinations occurred in the emergency room 87 times, and on the inpatient unit 168 times. Hepatomegaly was correctly diagnosed by medical students 44% of the time, by residents 73% of the time and by staff 68% of the time. This difference was not statistically significant (*p* = 0.34). For the residents, R1s diagnosed it correctly 77% of the time, R2s 54%, R3s 81% and R4s 75% of the time (*p* = 0.69). In terms of location, hepatomegaly was correctly diagnosed 67% of the time in the emergency department and 70% of the time on the inpatient unit (*p* = 0.92). Hepatomegaly was under-diagnosed (physical examination normal and ultrasound documented enlarged) in 48 of 254 examinations or 19% of the time. It was over-diagnosed (physical exam noted as enlarged and ultrasound examination normal) in 33 of 254 cases or 13% of the time.

## Discussion

Pediatric residents had the best ability to clinically detect hepatosplenomegaly, and were better than both staff and medical students at this skill. The finding that residents were better than medical students is similar to the finding in other studies that, as training progressed from medical student to resident, there was improvement in many clinical skills including the ability to detect cardiac murmurs,^5^,^6^ ECG interpretation,^8^,^21^ chest x ray interpretation,^9^ otoscopic interpretive skills,^22^ and blood culture acquisition.^10^ The current study is the first to show that the clinical skill declined in comparing resident performance to staff performance. Although this decline was not significantly different from the resident level, one would expect the staff ability to be higher than the resident ability, since many other studies have shown that, as clinicians become more focused on a particular field, their abilities improve. This is the case for blood culture acquisition,^10^ cardiac examination^1^ and cardiac murmur detection.^12^

Splenomegaly detection improved as the residents progressed through their training, which is in concordance with published studies of many clinical skills.^5^–^12^ Interestingly, hepatomegaly detection was better in the first and third years of training than in the second and fourth years of training, for which no clear reasoning is available.

Patients presenting with leukemia are for the most part first examined in the emergency department and then examined again on the inpatient unit. Thus the inpatient unit examination may be influenced by previous examinations that occurred in the emergency department, thus causing an increased detection in this setting. As well, the majority of patients admitted would have had a presumptive diagnosis of leukemia, so the examiners would potentially have had an increased index of suspicion for the finding of hepatosplenomegaly given its high prevalence at diagnosis of leukemia. This, however, was not the case in this study, with splenomegaly diagnosed correctly 76% of the time in the emergency department and 69% on the inpatient unit, and hepatomegaly 67% of the time in the emergency department and 70% on the inpatient unit. Thus the location did not affect the detection of these physical abnormalities, and this implies that the clinical detection skills were not influenced by previous examinations.

In the majority of cases in a teaching hospital, as ours is, patients are first examined by medical students, followed by residents and then staff, so as the patients are examined by subsequent clinicians, the initial findings may have been known by the examiner, again influencing their physical examination findings. Thus, one would expect that the staff findings would be the most accurate, reflecting not only their findings, but the findings of the trainees who examined the patient prior to them. Thus the finding that the staff detection was lower than the resident detection is surprising, but does suggest that previous findings are not influencing the subsequent examinations.

This patient population was chosen due to the high prevalence of hepatosplenomegaly in children newly diagnosed with leukemia and the fact that the majority of children undergo ultrasound examination, allowing a definitive diagnosis of hepatosplenomegaly. There was no control group that did not have a high prevalence, thus a high index of suspicion was present in those conducting the physical exam. This is a limitation of this study and may represent a higher detection level. Another limitation is that there are no evidence-based guidelines for assessing competency in the detection of hepatosplenomegaly on clinical exam, something which would be useful to study further.

## Conclusions

Overall, the level of training did influence the detection of hepatosplenomegaly with residents being more skilled than medical students at this skill, although the difference among the students, residents and staff was not significantly different. Surprisingly, residents were more skilled than staff physicians at this skill, which suggests that CME courses on physical examination skills may be of benefit to practicing physicians.

## Figures and Tables

**Table 1:** Patient characteristics.

Patient Characteristic	Number (Total =105)	Percentage
Male Gender	54	51.4
Acute Lymphoblastic Leukemia (ALL)	95	90.5
Acute Myeloid Leukemia (AML)	10	9.5
Body Mass Index > 18.5	34	32.3
Splenomegaly on Ultrasound	67	63.8
Hepatomegaly on Ultrasound	90	85.7
Age	Mean 7.53 years	Range 0.2–17.1 years

## References

[1] Lamb PM, Lund A, Kanagasabay RR, Martin A, Webb JA, Reznek RH (2002). Spleen size: how well do linear ultrasound measurements correlate with three-dimensional CT volume assessments. Br J Radiol.

[2] Homeida M, Roberts CJC, Halliwell M, Jackson L, Read AE (1976). Ultrasonic measurement of liver size. BMJ.

[3] Kratzer W, Fritz V, Mason RA, Haenle MM, Kaechele V (2003). Factors affecting liver size – a sonographic survey of 2080 subjects. J Ultrasound Med.

[4] Tamayo SG, Rickman LS, Mathews WC, Fullerton SC, Bartok AE, Warner JT (1993). Examiner dependence on physical diagnostic tests for the detection of splenomegaly: a prospective study with multiple observers. J Gen Intern Med.

[5] Vukanovic-Criley JM, Hovanesyan A, Criley SR, Ryan TJ, Plotnick G, Mankowitz K (2010). Confidential testing of cardiac examination competency in cardiology and noncardiology faculty and trainees: a multicenter study. Clin Cardiol.

[6] Favrat B, Picoud A, Jaussi A (2004). Teaching cardiac auscultation to trainees in internal medicine and family practice: does it work?. BMC Med Educ.

[7] Sinha P, Hogle NJ, Fowler DL (2008). Do the laparoscopic skills of trainees deteriorate over time?. Surg Endosc.

[8] Hoyle RJ, Walker KJ, Thomson G, Bailey M (2007). Accuracy of electrocardiogram interpretation improves with emergency medicine training. Emerg Med Australas.

[9] Eisen LA, Berger JS, Hegde A, Schneider RF (2006). Competency in chest radiography. a comparison of medical students, residents, and fellows. J Gen Intern Med.

[10] Parada JP, Schwartz DN, Schiff GD, Weiss KB (2005). Effects of type and level of training on variation in physician knowledge in the use and acquisition of blood cultures: a cross sectional survey. BMC Infect Dis.

[11] Kramer AW, Jansen KJ, Dosman H, Tan LS, van der Vleuten CP, Grol RP (2003). Acquisition of clinical skills in postgraduate training for general practice. Br J Gen Pract.

[12] Mahnke CB, Nowalk A, Hofkosh D, Zuberbuhler JR, Law YM (2004). Comparison of two educational interventions on pediatric resident auscultation skills. Pediatrics.

[13] Merrick HW, Nowacek GA, Boyer J, Padgett B, Francis P, Gohara SF (2002). Ability of the objective structured clinical examination to differentiate surgical residents, medical students, and physician assistant students. J Surg Res.

[14] Wu EH, Fagan MJ, Reinert SE, Diaz JA (2007). Self-confidence in and perceived utility of the physical examination; a comparison of medical students, residents and faculty internists. J Gen Intern Med.

[15] Cerilli GJ, Merrick HW, Staren ED (2001). Objective Structured Clinical Examination technical skill stations correlate more closely with postgraduate year level than do clinical skill stations. Am Surg.

[16] Filippi CG, Meyer RE, Cauley K (2010). The misinterpretation rates of radiology residents on emergent neuroradiology magnetic resonance (MR) angiogram studies: correlation with level of residency training. Emerg Radiol.

[17] Leon C, Asif A (2008). Physical examination of arteriovenous fistulae by a renal fellow: does it compare favourably to an experienced interventionalist?. Semin Dial.

[18] Margolis SA, Nilsson KA, Reed RL (2003). Performance in reading radiographs: does level of education predict skill?. J Contin Educ Health Prof.

[19] Baxter AL, Fisher RG, Burke BL, Goldblatt SS, Isaacman DJ, Lawson ML (2006). Local anesthetic and stylet styles: factors associate with resident lumbar puncture success. Pediatrics.

[20] Brunswick JE, Ilkhanipour K, Fuchs S, Seaberg D (1996). Emergency medicine resident interpretation of pediatric radiographs. Acad Emerg Med.

[21] Snyder CS, Bricker JT, Fenrich AL, Friedman RA, Rosenthal GL, Johnsrude CL (2005). Can pediatric residents interpret electrocardiograms?. Pediatr Cardiol.

[22] Jones WS, Kaleida PH, Lopreiato JO (2004). Assessment of pediatric residents’ otoscopic interpretive skills by videotaped examinations. Ambul Pediatr.

